# Facial Skin Manifestation of a Destructive, Nonkeratinizing Squamous Cell Carcinoma

**DOI:** 10.7759/cureus.66797

**Published:** 2024-08-13

**Authors:** Vixey Silva, Richard G Bindernagel, Melissa Cheng, Richard Miller

**Affiliations:** 1 Dermatology, HCA Healthcare/University of South Florida (USF) Morsani College of Medicine: Largo Medical Center Program, Largo, USA; 2 Dermatology, Western University of Health Sciences, Pomona, USA

**Keywords:** aggressive destructive lesion, p16 testing, squamous cell carcinoma, nasopharyngeal biopsy, hpv-related oropharyngeal cancer

## Abstract

Nonkeratinizing squamous cell carcinoma (NKSCC) of the sinonasal tract is a rare malignancy that infrequently presents with cutaneous extension. This case describes an unusual instance of extensive facial skin involvement in an elderly male with multiple comorbidities, diagnosed with a biopsy-proven NKSCC. The tumor was p16 positive, suggesting an HPV-related etiology. This case emphasizes the critical role of dermatologists in the early diagnosis and multidisciplinary management of such aggressive tumors. It highlights the necessity of including NKSCC in the differential diagnosis of destructive facial tumors. Dermatologists should remain vigilant for rare presentations and employ early biopsy and histopathological examinations to facilitate prompt multidisciplinary intervention. Recognizing p16 positivity can indicate a human papillomavirus (HPV)-related etiology, potentially influencing prognosis and management strategies.

## Introduction

Nonkeratinizing squamous cell carcinoma (NKSCC) is a sinonasal malignancy that is infrequently encountered among dermatologists. While documented cases demonstrate extensive local invasion involving nasofacial bones and intracranial infiltration, instances of cutaneous extension are exceptionally rare [1,2]. The differential diagnosis of midline facial destructive lesions includes but is not limited to, squamous cell carcinoma, basal cell carcinoma, rhinoscleroma, and Wegener's granulomatosis. The prognosis of sinonasal NKSCC is influenced by factors such as tumor location, tumor extension, stage at diagnosis, patient age, and lifestyle factors, including smoking and alcohol use [1,2]. Additionally, nonspecific symptoms leading to delayed diagnosis, advanced disease stages at presentation, and the tumor's proximity to vital structures can all worsen the prognosis [2].

Tumor immunohistochemistry profiling, particularly p16 positivity, plays a crucial role in diagnosing NKSCC and may indicate an etiologic role of human papillomavirus (HPV) [3]. A multidisciplinary approach involving dermatologists, oncologists, otolaryngologists, radiologists, and pathologists is paramount for patients with these tumors [4]. This case aims to present the unique clinical aspects and diagnostic considerations of NKSCC while emphasizing the importance of collaborative efforts for optimal patient outcomes.

## Case presentation

**Figure 1:**
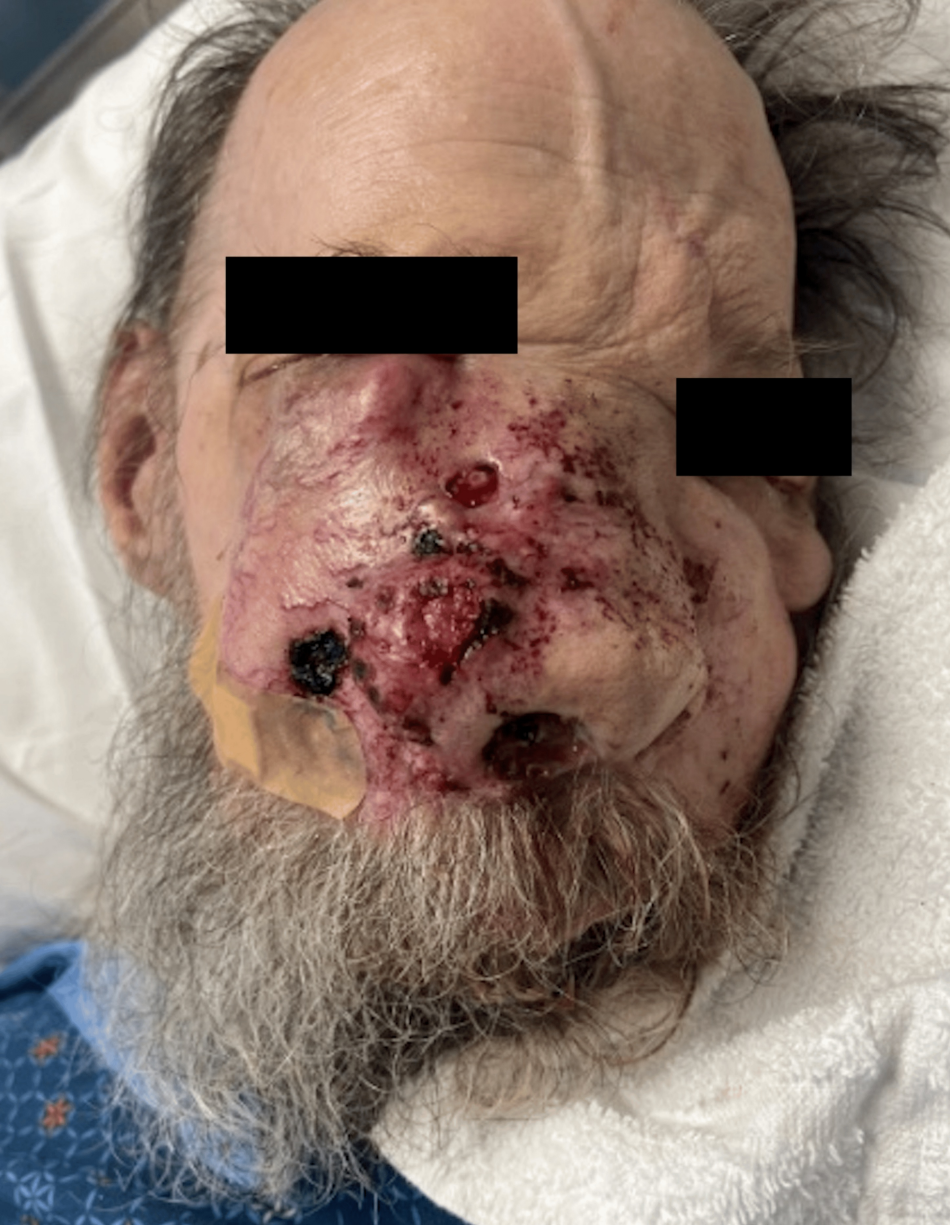
Erythematous, ulcerated plaque with multiple nodules on the central face, disrupting normal facial anatomy

**Figure 2:**
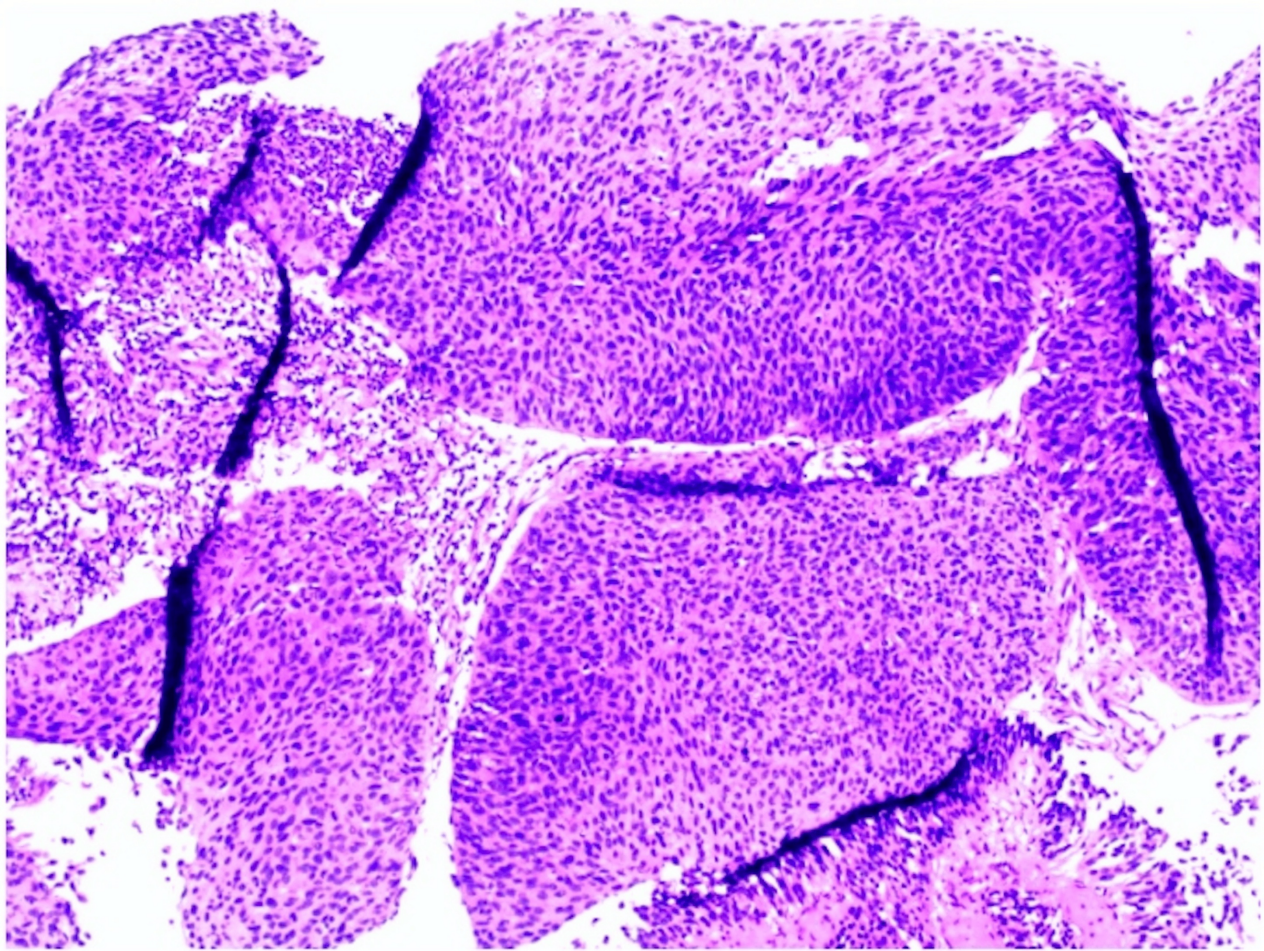
Hematoxylin and eosin (H&E) showing nests of uniform cells with abundant mitosis pushing on surrounding stroma

**Figure 3:**
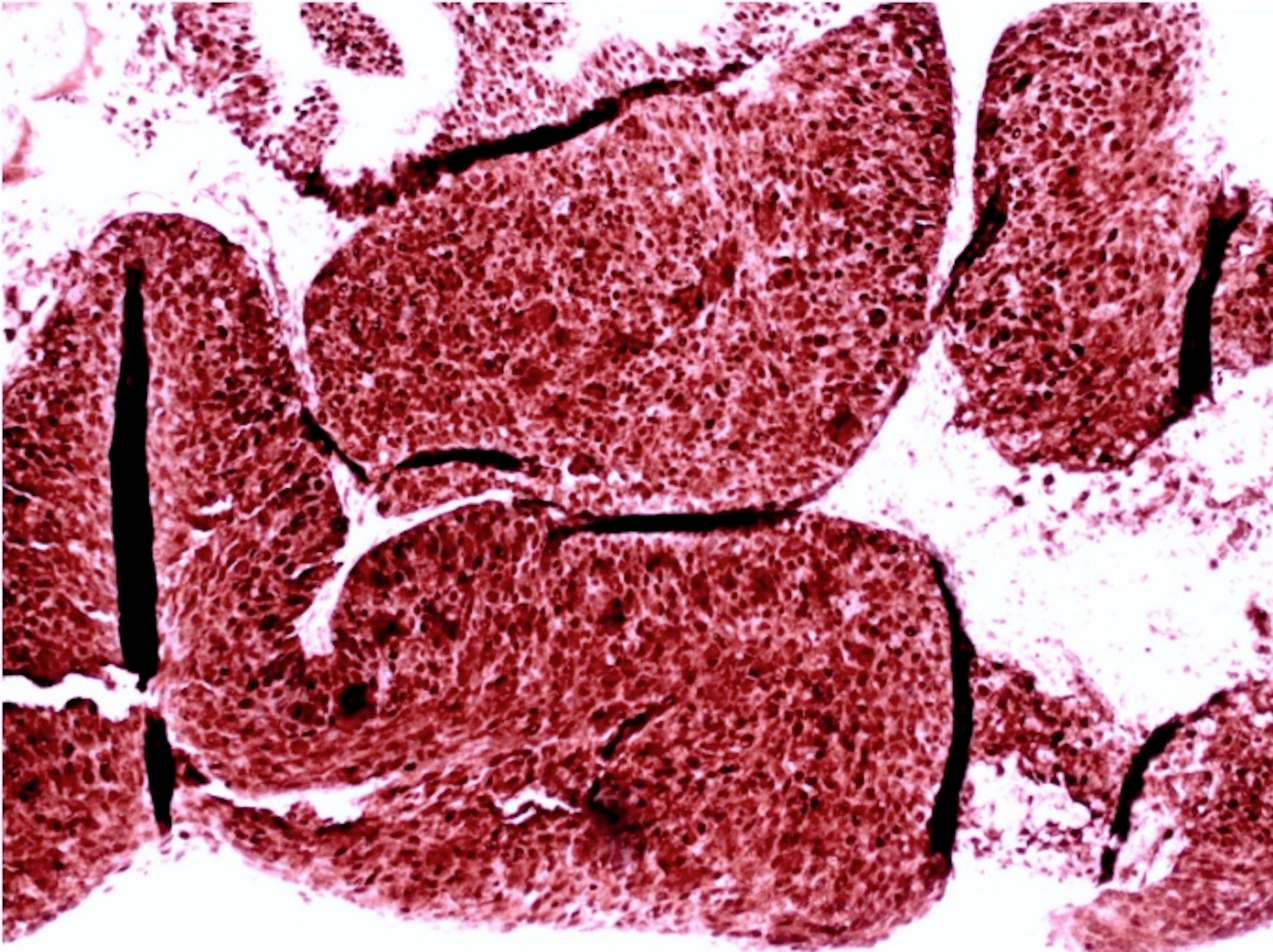
Immunohistochemistry of the tumor demonstrating diffuse positivity for p16

A 79-year-old male with a past medical history of diabetes mellitus type II, hypertension, and alcohol use disorder presented to the hospital with a large, rapidly growing, destructive facial mass. Upon examination, the patient appeared cachectic. He had a large ulcerative plaque with multiple nodules and overlying hemorrhagic crust located on his central face involving his eyes, nose, and mouth (Figure 1). Oral examination revealed an ulcerative plaque located on the gingiva and hard palate. Interventional radiology performed a core biopsy from the paranasal sinuses, which revealed a high-grade, invasive, non-keratinizing squamous cell carcinoma characterized by nests of uniform cells with abundant mitosis pushing on the surrounding stroma as seen on histology (Figure 2). Immunohistochemistry of the tumor demonstrated positivity for p63, p40, CK5/6, and p16 (Figure 3) with a high proliferative index (>80%) as evidenced by Ki-67 staining, all suggestive of NKSCC. Computed tomography imaging revealed a large, lobulated facial mass with destruction of the facial bones and sinus walls as well as orbital invasion. Treatment of the NKSCC was precluded by the patient’s demise secondary to complications thought to be unrelated to this diagnosis.

## Discussion

Common cutaneous tumors, such as basal cell carcinoma (BCC) and squamous cell carcinoma (SCC), often affect the head and neck and can display aggressive behavior [5]. Dermatologists should be aware of potential rare mimickers, such as NKSCC, which is found in the paranasal and nasal cavities arising from the surface epithelium lining the nasal cavity. Typical presenting symptoms for NKSCC include epistaxis, nasal stuffiness, swelling, or nasal ulcers [2]. Although local bone and intracranial infiltration has been documented, literature has rarely reported extension to the skin, as seen in our elderly patient. Because skin extension is uncommon, when skin manifestations are present, it typically represents an advanced disease state. Nonkeratinizing squamous cell carcinoma warrants consideration in the differential diagnosis of destructive facial tumors.

NKSCC is seen in males more than females, with most patients' age of onset being greater than 55 years [1]. It is debated whether tobacco and alcohol use and environmental and occupational exposures predispose patients to an increased risk of NKSCC [1]. Twenty percent to 25% of NKSCC cases are positive for HPV, confirmed by immunohistochemistry [1]. P16 is markedly positive in these tumors and can be used as a surrogate marker for HPV etiology. Increased p16 expression may represent downstream effects of HPV transcription proteins as was observed in our patient [6]. Positive p16 expression has also been associated with better outcomes and improved survival [4].

Our patient presented with a rapidly enlarging, non-healing, necrotic plaque, which under any circumstance should warrant a biopsy. Histological examination distinguishes NKSCC from its keratinizing counterpart, revealing large nests with abundant mitosis and central necrosis, which push surrounding structures out of the way. Additionally, these tumors lack keratin and show minimal squamous cell maturation. In contrast, keratinizing SCC (KSCC) nests are characterized by their infiltrative nests and the presence of keratin. These cells will have a more abundant, eosinophilic cytoplasm and exhibit diffuse squamous maturation [7]. Although NKSCC generally has a better prognosis than KSCC, its significance diminishes as tumors advance. Unfortunately, most nasopharyngeal carcinomas present at locally advanced stages, with a 25% five-year disease-specific survival rate in stage III or IV disease [8]. Therefore, with high clinical suspicion and an accurate understanding of histology, a dermatologist can aid in the detection of NKSCC and prompt referral to the necessary specialists.

## Conclusions

Nonkeratinizing squamous cell carcinoma of the sinonasal tract is a rare malignancy that can present with extensive cutaneous involvement. This case highlights the importance of considering NKSCC in the differential diagnosis of destructive facial tumors. Dermatologists play a crucial role in early detection through vigilant examination and prompt biopsy. The p16 positivity observed in this case suggests a potential HPV-related etiology, which may influence prognosis and treatment strategies. This presentation underscores the need for a multidisciplinary approach involving dermatologists, oncologists, otolaryngologists, radiologists, and pathologists to ensure optimal patient outcomes. Early recognition and collaborative management are essential in addressing the challenges posed by this aggressive malignancy.
